# Meterological Report for December, 1880

**Published:** 1881-01

**Authors:** 


### METEOROLOGICAL REPORT FOR DECEMBER, 1880.

| RAIN. | | Mean daily Barometer. | Mean daily Thermometer | Mean daily Humidity. | Maximum Thermometer | Minimum Thermometer | Prevailing direction of Wind. | Maximum hourly velocity of Wind, (Daily.) |
|---|---|---|---|---|---|---|---|---|
| DATE | IN. | | | | | | | |
| 1 | 1.01 | 29.959 | 59.5 | 84.7 | 64 | 50 | S. W. | ......... |
| 2 | 01 | 30 131 | 52.5 | 71.3 | 59 | 49 | N. W. | ......... |
| 3 | 0 | 30.158 | 48.2 | 67.0 | 55 | 37 | E. | ......... |
| 4 | 52 | 29.996 | 48.2 | 90.0 | 50 | 40 | E. | ......... |
| 5 | 1.33 | 29.825 | 59.2 | 92.3 | 69 | 48 | S. W. | ......... |
| 6 | 36 | 30.109 | 43.7 | 51.0 | 56 | 36 | N. W. | ......... |
| 7 | 0 | 30.358 | 33.0 | 53.7 | 42 | 24 | N. W. | ......... |
| 8 | 0 | 30.189 | 48,7 | 53 3 | 56 | 34 | S. W. | ......... |
| 9 | 0 | 30.473 | 30.7 | 55.0 | 42 | 24 | N. W. | ......... |
| 10 | 0 | 30 562 | 33.7 | 45.3 | 42 | 21 | N. W. | ......... |
| 11 | 0 | 30.295 | 42.5 | 33.3 | 50 | 25 | S. W. | ......... |
| 12 | 02 | 29.955 | 47.7 | 42 7 | 55 | 33 | S. W. | ......... |
| 13 | 0 | 29 956 | 51.7 | 63.0 | 59 | 37 | S. W. | ......... |
| 14 | 02 | 29.895 | 56.7 | 82.3 | 60 | 42 | S. W. | ......... |
| 15 | 0 | 29.885 | 59.5 | 73.0 | 65 | 50 | S. W. | ......... |
| 16 | 18 | 29.769 | 65.0 | 83.0 | 70 | 60 | S W. | ......... |
| 17 | 78 | 29.817 | 59.7 | 90.0 | 66 | 56 | Calm. | ......... |
| 18 | 03 | 29 881 | 55.2 | 69 3 | 64 | 49 | N. W. | ......... |
| 19 | 23 | 29.873 | 41.2 | 75.0 | 50 | 37 | N. E. | ......... |
| 20 | 07 | 29 912 | 36.5 | 81.3 | 43 | 33 | N. W. | ......... |
| 21 | 0 | 30.135 | 33.0 | 79.3 | 36 | 30 | N. W. | ......... |
| 22 | 0 | 30.249 | 33.7 | 73.7 | 40 | 30 | N. W. | ......... |
| 23 | 0 | 30.199 | 43.2 | 63.3 | 50 | 31 | S E. | ......... |
| 24 | 0 | 30.130 | 44.5 | 66.0 | 53 | 36 | S. E. | ......... |
| 25 | 76 | 29.973 | 39.2 | 87.7 | 46 | 35 | N. E. | ......... |
| 26 | 0 | 30.045 | 34.7 | 70.7 | 45 | 33 | N. W. | ......... |
| 27 | 0 | 30.051 | 34.0 | 64.7 | 38 | 29 | N. W. | ......... |
| 28 | 0 | 29.995 | 34.7 | 67.7 | 41 | 30 | N. W. | ......... |
| 29 | 28 | 30.136 | 12.5 | 76.0 | 34 | 6 | N W. | ......... |
| 30 | 0 | 30.355 | 74.7 | 44 7 | 26 | 1 | W. | ......... |
| 31 | 0 | 30.326 | 23.0 | 50.0 | 34 | 4 | N. W. | ......... |
| Sums | | ...... .. | ...... | ...... | ... | ... | ......... | ......... |

